# Facile synthesis of nano-sized CuFe_2_S_3_: morphology and diverse functional tuning and crystal growth mechanism exploring

**DOI:** 10.1093/rb/rbx006

**Published:** 2017-06-05

**Authors:** Xiao Zhang, Huan Zhao, Yuda Zhu, You Yang, Dongli Jiang, Xiaoqin Chen, Jing Sun, Jiaoming Luo, Bing Cai, Hongsong Fan

**Affiliations:** 1 Analytical & Testing Center, Sichuan University, Chengdu, Sichuan 610064, China; 2 National Engineering Research Center for Biomaterials, Sichuan University, Chengdu, Sichuan 610064, China

**Keywords:** nanobiomaterials, materials synthesize

## Abstract

Ternary chalcogenide compounds are such promising and have been used for much practical applications. As a sort of these compounds, cubanite (CuFe_2_S_3_) possess some unique properties which can be used in different fields. In our study, we developed a facile one pot synthesis of CuFe_2_S_3_ nanocrystals (NCs) at a low reaction temperature, and achieved a morphology and phase composition tuning of the NCs through changing a variety of precursors and surfactants, meanwhile improved their magnetism and optical properties. Eventually, well-ordered and ‘nano-brick’ like CuFe_2_S_3_ NCs were obtained and showed best magnetism and near-infrared fluorescence properties. Furthermore, the NCs were proved with good biocompatibility and possibility for cell labeling. This kind of materials with lower toxicity and potential of magnetic is bound to remedy the defects of photoluminescence quantum dots (QDs) and be with higher potential in the field of biological diagnosis and multi-functional system construction.

## Introduction

Ternary chalcogenide compounds have attracted an extensive attention owe to their unique physical and chemical properties, such as magnetism and photoelectric properties [1–7]. Furthermore, with near-infrared fluorescence and low toxicity, ternary luminescence nano-materials also have shown great potential as an alternative to the traditional binary quantum dots in the field of bioimaging and multifunctional system construction [8,9]. Among them, ternary copper iron sulfide compounds such as chalcopyrite and cubanite (CuFe_2_S_3_) are unique candidates, owing to their good biocompatibility and magnetism potential. However, to the best of our knowledge, the majority of former reports usually focus on the crystal and electronic structure of chalcopyrite, and only a few reports referred the synthesis of CuFe_2_S_3_ [10–13].

Natural mineral CuFe_2_S_3_ has been acknowledged to be with orthorhombic structure [14–16], in which the cations are tetrahedrally coordinated by S atoms, forming an approximately hexagonal packing. The Fe^2+^ and Fe^3+^ ions share the adjacent edges of tetrahedral and there is a rapid electron transfer between them [17,18]. However, in the previous reports, it has been proven that the CuFe_2_S_3_ will transform to a cubic form at high temperature [19–21]. The crystal structure of this cubic CuFe_2_S_3_ is based on a cubic close-packed matrix of sulfur atoms, wherein the metal atoms are located in the tetrahedral interstices, furthermore, the ferrous, iron and cuprous ions are randomly distributed on these cation sites [15,20]. This special structure endows cubic CuFe_2_S_3_ with unique photoelectricity and magnetic properties, which play a crucial role in biological application. However, most of the existed reports on CuFe_2_S_3_ are about crystal in bulk, either using naturally existing or synthetic single crystal or polycrystalline compounds. There have been very few studies on the synthesis and characterization of nano-sized crystalline CuFe_2_S_3._ More studies are further needed to investigate not only the synthesis but also the modulation of CuFe_2_S_3_ crystal morphology, luminescence and magnetic properties. In most cases, the synthesis of CuFe_2_S_3_ usually needs strict condition with a high temperature exceeding 200°C. Therefore, it is a challenge to develop a facile and mild strategy for fabricating nano-sized CuFe_2_S_3_ under laboratory conditions and study their prospect in biological application.

In this study, CuFe_2_S_3_ NCs with uniform and small size, as well as magnetism and near-infrared fluorescence properties would be prepared. A facile and mild strategy to fabricate CuFe_2_S_3_ NCs under a lower reaction temperature (180°C) was developed. Meanwhile, the morphology and physical properties of these NCs have been finely modulated by changing the reactants. Furthermore, cubic CuFe_2_S_3_ NCs with magnetism and near-infrared fluorescence properties showed great potential application on cell labeling. Our work is bound to encourage further exploration in the synthesis of nano-sized CuFe_2_S_3_ under laboratory conditions and expand their multifunctional application in the biological field.

## Experimental

### Reagents and materials

Iron (III) chloride hexahydrate (FeCl_3_·6H_2_O, ACS), iron(III) acetylacetonate [Fe(III)(acac)_3_, 98%], copper (II) chloride dihydrate (CuCl_2_·2H_2_O, 99.99%), copper(II) acetylacetonate [Cu(II)(acac)_2_, 97%], 1-dodecanethiol (DT, 98.0%), sodium diethyldithiocarbamate trihydrate (DDTC, 99.0%), thiourea (CH_4_N_2_S, 99.0%), oleylamine (OAM, 80–90%) and 1-octadecene (ODE, >90.0%) were purchased from Shanghai Aladdin Industrial Corporation, China. Oleic acid (OA, >85.0%) were purchased from Tokyo Chemical Industry Co. Absolute ethanol was purchased from Kelong Chemical Reagent Factory, China.

### Synthesis of CuFe_2_S_3_ nanocrystals (CuFe_2_S_3_ NCs)

**Figure 1:**
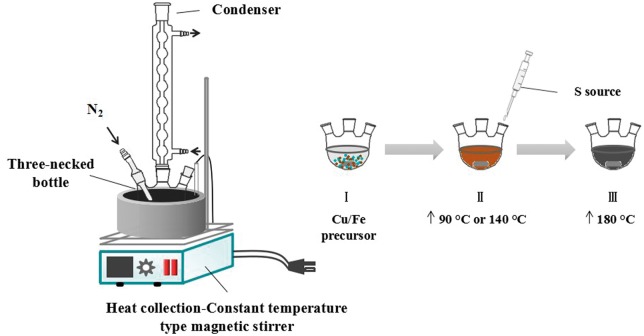
the One pot synthesis of CuFe_2_S_3_ nanocrystals

CuFe_2_S_3_ NCs were prepared through a one pot method. The device diagram of experimentation is shown in Fig. 1. For a typical synthesis, 0.1705 g CuCl_2_·2H_2_O and 0.2703 g FeCl_3_·6H_2_O were added into a mixture of 12 ml OA and 18 ml DT in a 100 ml three-necked round-bottom flask. The flask was put into a constant temperature heating magnetic stirrer with oil bath heating (140°C) under N_2_ flow until reactants were fully dissolved. The S-precursor suspension was freshly prepared by mixing 0.1522 g of thiourea with 6 ml of DT under magnetic stirring in air and preheated to 100 °C. Next, the S-precursor solution was transferred into a syringe (equipped with a large needle) and injected quickly into the flask at 140°C. The temperature of mixture was further quickly raised to 180°C and kept for 15 min. To terminate the reaction, the flask was quickly transferred to a cold-water bath. The resulting nanocrystals were separated from the dispersion solution by centrifugation (4000 rpm, 5 min) and washed by ethanol for several times to remove the impurities. The obtained products were dispersed in absolute ethanol for further characterization.

In order to achieve the structure and morphology modulation, and further optimize the luminescence and magnetism properties of CuFe_2_S_3_ NCs, different iron and copper precursors, sulfur sources and surfactants were chosen in the following experiments. The detailed preparation conditions are shown in Table 1.

**Table 1:** The synthesis of CuFe_2_S_3_ nanocrystals

Method	Reactant	Solvent	S source	Temperature (°C)
S1	Cu_(II)_(acac)_2_	OA + DT	DDTC	140 →180
	Fe_(III)_(acac)_3_			
S2	Cu_(II_)(acac)_2_	OA + ODE + OAM	Thiourea	140 →180
	Fe_(III)_(acac)_3_			
S3	CuCl_2_·2H_2_O	OA + ODE + OAM	Thiourea	140 →180
	FeCl_3_·6H_2_O			
S4	CuCl_2_·2H_2_O	OA + DT	Thiourea	90 →180
	FeCl_3_·6H_2_O			
S5	CuCl_2_·2H_2_O	OA + DT	Thiourea	140 →180
	FeCl_3_·6H_2_O			

### Material characterization

X-ray diffraction (XRD): The prepared samples were dried in a drying oven under 70°C, and the crystalline structure were measured on an X-ray diffractometer (Panalytical Empyrean), at 45 kV and 40 mA, for monochromatized Cu Kα (λ = 1.5418 Å) radiation.

Raman spectra (RM): The Raman spectrum of dry sample was recorded on a Confocal Laser MicroRaman Spectrometer (LABRAM-HR) with 514.5 nm radiations from a 10 mW argon ion laser at room temperature.

X-ray photoelectron spectroscopy (XPS): The samples were re-dispersed in absolute ethanol with a sufficient ultrasonic oscillating, then dropped on a silicon slice. The XPS measurements were carried out on a Kratos Axis Ultra DLD spectrometer equipped with a monochromatic Al Ka X-ray source.

Transmission electron microscope (TEM): The samples were re-dispersed in absolute ethanol under sufficient ultrasonic oscillating, then dropped on a copper net coating with a carbon film. The morphology and microstructure of CuFe_2_S_3_ NCs were monitored using high-resolution transmission electron microscopy (HRTEM) on TecnaiG2F20S-TWIN microscope at 200 KV.

Magnetism measurement: The magnetic measurements of dry CuFe_2_S_3_ NCs were performed by using a superconducting quantum interference magnetometer (MPMS-XL-7, Quantum Design) at both room temperature (300 K) and low temperature (3 K).

Photoluminescence characterization: The samples were dispersed in absolute ethanol with a sufficient ultrasonic oscillating, then 1 ml of the solution was taken. The fluorescence emission of CuFe_2_S_3_ NCs was collected by using a fluorescence spectrometer (F-7000, Hitachi), using an emission wavelength at 500 nm.

### Potential application of CuFe_2_S_3_ NCs

Cytotoxicity study: The cytotoxicity of CuFe_2_S_3_ NCs against human osteosarcoma cell line MG63 cells was studied using standard methyl thiazolyltetrazolium (MTT, Sigma Aldrich) assay. MG63 cells were dispensed onto a 24-well plate at a density of 1 × 10^4^ cells per well. After culturing 24 h for cell attachment, the media were taken out from the wells, followed by washing three times with PBS, and then incubated with various concentrations of CuFe_2_S_3_ NCs (30, 90 and 150 µg/mL). After further incubation for 1, 3 and 5 d, the solutions were changed with fresh DMEM culture medium, cell viability was studied by standard methyl thiazolyltetrazolium assay.

Cell labeling: The cell-labeling capacity of CuFe_2_S_3_ NCs was investigated as follows: MG63 cells were seeded onto a 24-well plate at a density of 3 × 10^4^ cells per well in a 24-well plate, and cultured in the culture box (CO_2_ 5%, 37 °C) for 24 h before adding the NCs. After cell attachment, the media were taken out from the wells, followed by washing three times with PBS. Cells were then incubated with CuFe_2_S_3_ NCs at a certain concentration (150 µg/mL) for 30 min. After cells were washed carefully using PBS to wipe off uninternalized nanoparticles and incubated for another 30 min, a confocal laser scanning microscopy (CLSM; LeicaSP5, Leica Microsystems, Germany) was acquired for cell imaging.

## Results

### Phase and chemical characterization

**Figure 2:**
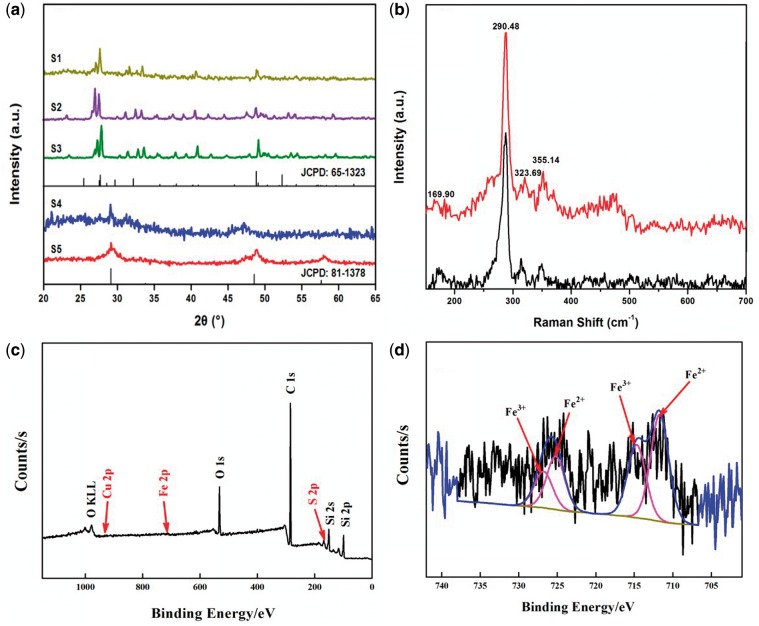
(**a**) XRD patterns of the prepared CuFe_2_S_3_ NCs with different conditions. (**b**) Raman spectra of S5 (red line) and corresponding standard spectrum of cubic cubanite (black line). (**c**) XPS survey spectrum and (**d**) Fe2p XPS spectra of S5

The phase composition of the samples was examined using XRD. As shown in Fig. 2a, the XRD spectra of S1, S2 and S3 have almost similar diffraction patterns which are corresponded to the orthorhombic CuFe_2_S_3_ (ICDD card, No. 65-1323), indicating the fabrication of relatively pure CuFe_2_S_3_ NCs with an orthorhombic structure by our facile and mild method. The sharp peaks and low background observed in S2 and S3 suggest a higher crystallinity of S2 and S3 than that of S1 with relative diffusion background. Contrarily, S4 and S5 show three different intense peaks at 2*θ *= 29.2°, 48.6° and 58.7° which are attributed to the cubic CuFe_2_S_3_ phase, oriented along the [111], [220] and [311] crystal planes, respectively [16]. These results indicate the successfully synthesis of cubic CuFe_2_S_3_ NCs, according to the standard spectra of cubic CuFe_2_S_3_ (ICDD card, No. 81-1378). Furthermore, compared with S1–S3, S4 and S5 presented relative disordered peaks which reveal a relative low crystalline of the samples, meanwhile S5 was with higher crystallinity than S4. These results reveal that nano-sized crystalline CuFe_2_S_3_ could be fabricated using our one pot method at a relative low temperature (180°C). The crystal structure of NCs could be adjusted from orthorhombic to cubic by controlling the reactants, surfactants and reaction conditions.

Figure 2b shows the Raman spectrum of CuFe_2_S_3_ nanocrystals of S5 at room temperature. According to the standard spectrum of cubic CuFe_2_S_3_, the stronger peak (290 cm^−1^) and three weaker peaks (frequency 169, 323 and 355 cm^−1^) are in good agreement with the standard spectrum, indicating that a relatively pure cubic CuFe_2_S_3_ has been obtained.

To further understand the nature of interactions among the atoms in product we have prepared, XPS measurements were undertaken. The XPS survey spectrum of the S5 NCs is shown in Fig. 2c, and the photoelectron peaks of Fe 2p is presented in Fig. 2d. In the survey and high-resolution spectra of S5, C 1 s (fitting element), Si 2 s and Si 2p peaks (silicon substrate) are also observed with the exception of the expected Cu 2p, S 2p and Fe 2p peaks. After peak fitting, four peaks appear in the high-resolution spectrum of Fe 2p, and two peaks appear at 711 and 725 eV, which can be attributed to the ionization of Fe 2p_3/2_ and Fe 2p_1/2_ electrons of Fe^2+^, furthermore it displays another two peaks at 714 and 726 eV corresponding to Fe 2p_3/2_ and Fe 2p_1/2_ electrons of Fe^3+^, respectively. These parameters almost coincide with the Fe^2+^ and Fe^3+^ surface species in the previous reports [22,23].

### Crystal morphology characterization

**Figure 3:**
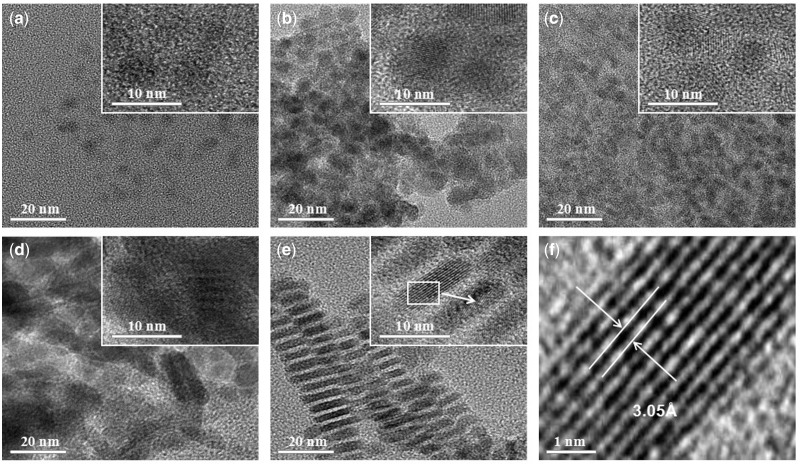
HRTEM Pictures of the synthesized CuFe_2_S_3_ NCs with different reaction conditions: (**a**) S1; (**b**) S2; (**c**) S3; (**d**) S4; (**e**) S5; (**f**) lattice fringes of the S5

Figure 3 displays the HRTEM images of the NCs of S1–S5, and insert maps show the enlarged images accordingly. As the images illustrate, the crystal morphology of samples varied from spherical to ‘brick’ like with the varying of the reaction precursors and surfactants from S1 to S5. Generally, the crystals of S1, S2 and S3 are all spherical-like nanocrystals with the average crystal sizes about 3, 4 and 4 nm, respectively (Fig. 3a–c). S2 and S3 show a relatively lower dispersity than S1. Furthermore, Fig. 3d and e shows that S4 and S5 have completely different crystal morphology in comparison with S1–S3. For S4, a mixed crystal morphology of spherical and ‘brick’ ones are presented. The diameter of spherical one is about 7 nm, while size of brick-like crystal is about 20 nm. The HRTEM imagine in Fig. 3d reveals that part of the lattice fringes of crystals could be observed. The brick-like ones have a better sharp fringe, and their crystal parameters are coincided with the cubic CuFe_2_S_3_ as well. In addition, for S5, all the NCs are of the shape of ‘brick’ and self-assemble in a certain orientation (Fig. 3e). These monodispersed nano-bricks are of 4 nm width and 8 nm length, and the distance between the nano-bricks are nearly the same about 2–3 nm. The enlarge scale of Fig. 3e clearly shows that each nano-brick has uniform morphology and good monodispersity. The lattice fringes of the crystals can be clearly seen with a lattice space about 3.05 Å between two adjacent lattice planes, which is consistent with the (111) plane (Fig. 3f) of cubic CuFe_2_S_3_ NCs, indicating that the crystallinity of S5 is much higher than S4.

### Magnetic characteristics of the CuFe_2_S_3_ NCs

**Figure 4:**
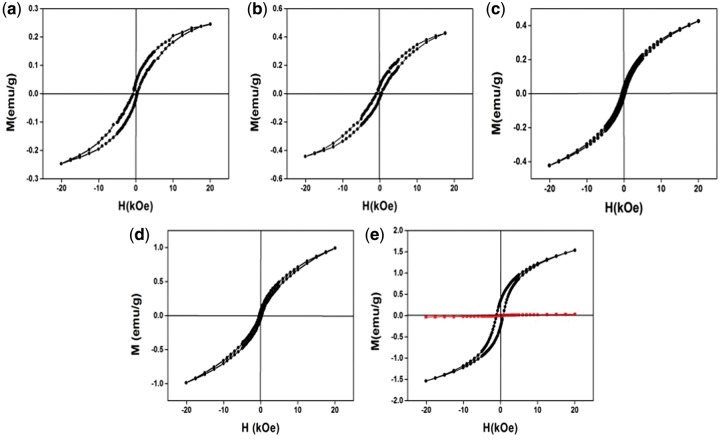
Magnetic characterization of the CuFe_2_S_3_ NCs prepared with different conditions under 3 K (**a**–**e**, black lines) and 300 K (e, red line)

The magnetism of the NCs was characterized by a superconducting quantum interference magnetometer, and the field-dependence magnetization curves M(H) are shown in Fig. 4. Under room temperature, all the NCs showed no magnetism (so that the data not shown here). Figure 4a–e shows the magnetization curves at 3 K of S1–S5, and the obvious coercivity could be observed in all the NCs, indicating the well magnetic property of CuFe_2_S_3_ NCs at low temperature. Furthermore, S4 and S5 show relatively higher magnetism than S1–S3, while S5 has the highest magnetism. These results indicate that the magnetism of CuFe_2_S_3_ NCs is dependent on the temperature. At low temperature, the whole NCs we synthesized possess the magnetism, and S5 shows the strongest magnetism among them all.

### Fluorescent characteristics of the CuFe_2_S_3_ NCs

**Figure 5:**
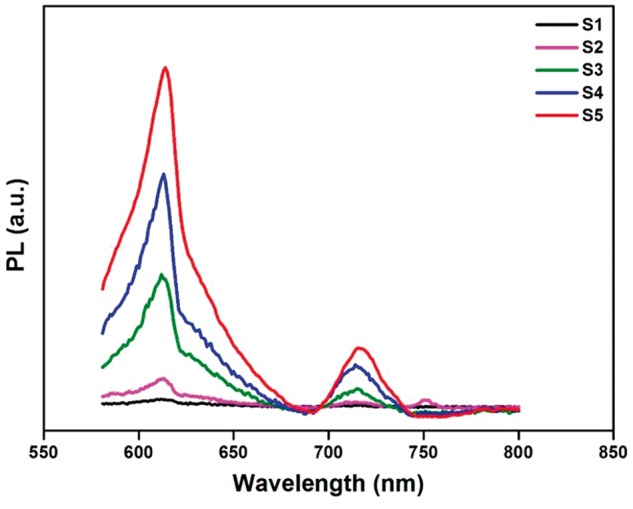
The PL spectra of CuFe_2_S_3_ NCs prepared with different conditions

The near-infrared fluorescence properties of CuFe_2_S_3_ were then studied. As shown in the PL emission spectra (Fig. 5), when the NCs were irradiated at 500 nm, a strong red emission peak at 614 nm and a weaken emission peak at about 715 nm were observed for all the NCs. The full width at half maximum (FWHM) of these two peaks are the same about 40 nm. Furthermore, the fluorescence intensity of the NCs increased with the order from S1 to S5, and S5 shows the strongest emission about 10 times than that of S1. These results demonstrate that the near-infrared fluorescence intensity of CuFe_2_S_3_ NCs varied with different precursors and reaction conditions. By judicious modulating the synthesis process, CuFe_2_S_3_ NCs with near-infrared emission would be obtained and show great potential for bioimaging.

### Potential evaluation of CuFe_2_S_3_ nanocrystals in bioimaging

**Figure 6:**
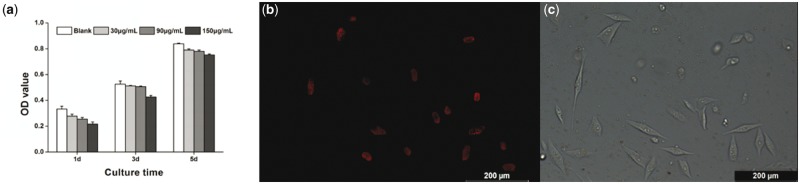
(**a**) Cytotoxicities of CuFe_2_S_3_ NCs with different concentrations to MG63 cells determined by the MTT cell proliferation assay (the results are obtained from three experiments with standard deviations) and the CLSM images of MG63 cells incubated with CuFe_2_S_3_ NCs under (**b**) fluorescent field and (**c**) bright field

Due to the stronger fluorescent intensity compared with other samples, S5 was chosen to evaluate the potential application of CuFe_2_S_3_ NCs in bioimaging. We first investigated their potential toxicity using human osteosarcoma cell line MG63 cells. For cytotoxicity test, MG63 cells were incubated with CuFe_2_S_3_ NCs of S5 at different concentrations for 1, 3 and 5 d, and the corresponding cell viability data were obtained by MTT assay. As shown in Fig 6a, the cells in all the groups proliferated dramatically along with time prolonged, although the samples incubated with NCs presented a little lower cellular survival than the control after 1 d of incubation. There is little difference in the percentage of viable cells between the experimental and control samples even at the highest concentration of 150 μg/mL in the following days, which suggested excellent cytocompatibility of the prepared CuFe_2_S_3_ NCs.

Encouraged by the strong near-infrared fluorescence and biocompatibility of the prepared CuFe_2_S_3_ NCs, we further investigated the potential of the prepared CuFe_2_S_3_ NCs for cell imaging. MG63 cells were first incubated with CuFe_2_S_3_ NCs of S5 for 30 min to label the cells, then the near-infrared fluorescence of intracellular NCs was observed by CLSM. Figure 6b is the fluorescent field of MG63 cells incubated with S5, compared with the bright field (Fig. 6c) of that. As CLSM images show, a red fluorescence signal can be observed inside the cells in Fig. 6b. Furthermore, the red fluorescence of CuFe_2_S_3_ NCs is homogeneously distributed in the cytoplasm instead of the cell nucleus. These results indicate that the prepared CuFe_2_S_3_ NCs with near-infrared fluorescence could penetrate into the living cells and be utilized for cell imaging.

## Discussion

As a typical representative of ternary chalcogenide compounds, CuFe_2_S_3_ with near-infrared fluorescence and good biocompatibility has attracted much attention in the field of photoelectricity and biomedical research. However, studies on the controllable synthesis of nanoscale CuFe_2_S_3_ are rarely reported. The above results demonstrate that nano-sized CuFe_2_S_3_ have been successfully fabricated using a facile and mild one pot synthesis approach. Most importantly, the phase composition, morphology and physical properties of prepared NCs can be modulated by changing the precursors and surfactants. Finally, CuFe_2_S_3_ NCs with good biocompatibility and near-infrared fluorescence has been proved to be potential for cell imaging.

### Growth mechanism of CuFe_2_S_3_ NCs based on reactants tuning

**Figure 7:**
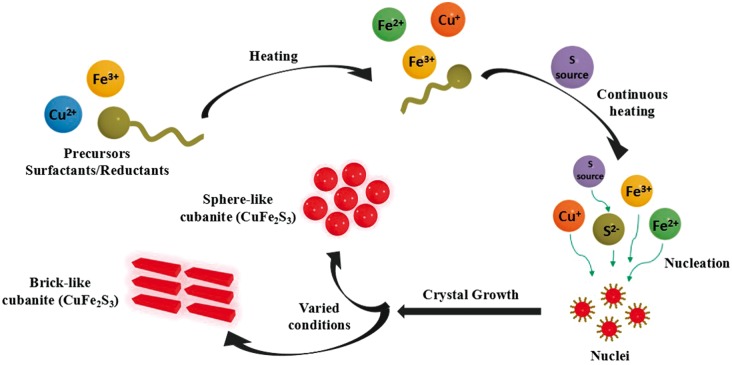
The growth mechanism of CuFe_2_S_3_ NCs

The possible growth mechanism is schematically illustrated in Fig. 7. Generally, the reactants of metal precursors, specific reducers and surfactants were mixed in the organic solvent to produce free Cu^2+^ and Fe^3+^, meanwhile some of these metal ions would be reduced to Cu^+^ and Fe^2+^ by the reducer in the system. At predetermined heating temperature, well-preheated S precursor was added, then S^2-^ ions were released and reacted with the free copper and iron ions in the solution, inducing a break precipitation of copper, iron and sulfur ions to form enormous CuFe_2_S_3_ nuclei [24]. Thanks to the strong surface activity, the free surfactant molecules in the solvent were probably to be attracted on the surface of the nuclei [25]. With continuous heating, the growing of the nuclei progressed with more free metal and sulfur ions continuously migrating to the surface of the nuclei. At the same time, the ions exchange and atom rearrangement both happened inside and on the surface of the crystals [26], resulting the variation of the crystal morphology, size, composition and crystallinity, until the stable CuFe_2_S_3_ NCs were formed. Obviously, the growth of CuFe_2_S_3_ NCs can be affected by the followings: (1) the nucleation and growth of CuFe_2_S_3_ nuclei are dependent on the producing speed of the free copper, iron and sulfur ions by reactants [27–29], (2) the ion exchange and atom rearrangement during crystal growth are affected by the properties of surfactant or solvent. Therefore, the phase composition and crystal morphology of CuFe_2_S_3_ NCs can be judiciously modulated under varied conditions.

As revealed in above results, although with different reactant precursors and surfactants, all the samples from S1 to S5 are pure CuFe_2_S_3_ NCs, suggesting the correctness of the proposed crystal growth mechanism and the efficiency of our one pot approach to prepare CuFe_2_S_3_ NCs. The totally different phase and crystal morphology of S4 and S5 compared with S1 to S3 should be mainly attributed to the varied conditions in the growth processes, including the metal precursors, surfactants and sulfur sources. First, the effect of metal precursors and varied S source on phase composition was revealed. As Fig. 2a–c shows, S1, S2 and S3 have the same phasic structure with similar spherical-like morphology. Comparing their respective reaction conditions in Table 1, it is easily inferred that different metal precursors (for S2 and S3) and varied S source (for S1 and S2) are not the decisive factors to affect crystal morphology and phase structure.

Second, the effect of the varied surfactants attributed to the differences in the crystal dispersity and size was studied. Although with similar phase composition in S1, S2 and S3, S1 showed a better particle dispersity, smaller crystal size and lower crystallinity than S2 and S3. Comparing the reactants, it can be inferred that the reaction using DT as surfactant (for S1) is inclined to produce smaller nano-crystals with better dispersity than the reaction using ODE + OAM as surfactants (for S2 and S3). With shorter molecular length and higher polarity, DT is more likely to move to the nuclei and bind with cations on the nuclei surface [30]. Therefore, DT molecules were more evenly distributed on the surface of CuFe_2_S_3_ nuclei, which in turn protect the nuclei from aggregation and restrict the continuous growth of crystals, producing the CuFe_2_S_3_ NCs with better dispersity, smaller size, but lower crystallinity.

In the following experiments, the combination using of DT as surfactant and thiourea as S source in S4 and S5 resulted in great change in crystal growth progress and the phase composition of final crystals. As above results show, S4 and S5 have the phase composition of cubic CuFe_2_S_3_ and brick-like crystal morphology, which were totally different with S1 to S3. This phenomenon may be explained as follows: Both DT and thiourea are strong reducers which resulted in supplying more Cu^+^, Fe^2+^ and S^2^^−^ in the system [31]. Therefore, in comparison with the S1 using DT as reductant and surfactant, DDTC as S source, and compared with S2 and S3 using thiourea as S source, ODE as reductant, there are more free ions for the deposition of ternary sulfide in S4 and S5. It should be noted that both the protection of DT and the selection of SH- on crystal orientation have significant impacts on the growth habit of nanocrystal [32,33], resulting in great change of phase composition and crystal morphology in the final products. On one hand, as above mentioned, DT with shorter molecular length moves easier to nuclei surface to form binding on the surface, on the other hand, it has been reported that such cations (Cu^+^, Fe^2+^, Fe^3+^) are especially sensitive to the sulfhydryl to form complexation, it would reduce not only the surface tension and surface energy but also the growing rates of each crystal planes, inducing the thoroughly change of crystal growing habit and crystal structure [34–38]. Therefore, the NCs in S4 and S5 would prefer to grow uniaxially along a few of the lattice planes, and formed the cubic CuFe_2_S_3_ with brick-like morphology. Furthermore, as the reduction reactions and change of surface planes growth rates are both energy required [39], meanwhile the motion rates of molecule and ions were slower at low temperature (90°C), S4 of adding S sources at lower temperature showed an incompletely change of crystal growth, forming lower crystalline cubic CuFe_2_S_3_ with mixing structure morphology of sphere-like and brick-like ones, as well as aggregated crystals. On the contrary, in S5 of combing DT and thiourea as at higher temperature (140°C), uniform and small CuFe_2_S_3_ with cubic structure were formed. Furthermore, this special habit of crystal growth is more or less to affect the atomic rearrangement, resulting in the reducing of crystallinity or leading to the formation of defects in crystals, thus S5 showed a relative lower crystallinity than S1 to S3.

The ordered arrangement of the brick-like crystals of S5 is related to their magnetism. As Fig. 4 shows, all the samples have obvious magnetic behavior at low temperature (3 K), especially S4 and S5 with cubic structure show higher magnetism, and the magnetism of S5 is the strongest. The strongest magnetism of S5 contributes to the lining of the single nano-brick with well-ordered alignment, in return this ordered alignment is possible to enhance the magnetism.

### The magnetism of CuFe_2_S_3_ NCs

The magnetism of CuFe_2_S_3_ mainly originates from the varied valence state of Fe atoms, as the copper ions in this compound are in the diamagnetic Cu^+^ (3d^10^) state [40]. The varied valence state of Fe atoms has also been proved by the XPS analysis above. Previous reports of Mössbauer spectra of CuFe_2_S_3_ NCs have shown that at room temperature the iron ions are in non-magnetic state [18]. However, at lower temperature, the cubic CuFe_2_S_3_ phase CuFe_2_S_3_ have stable charges of Fe^2+^ and Fe^3+^ in the tetrahedral sulfur sites, along with electron exchange between these ions, so the magnetization is observed [41]. Furthermore, as above discussed, S4 and S5 with more Fe^2+^ produced by stronger reduction would accelerate atomic exchange and varied ratio of two valences stated iron ions, resulting in stronger magnetism. Another reason for the producing of magnetization is the presence of Fe–S type of magnetism impurities in the CuFe_2_S_3_ crystals [42]. It has been discussed above, more S^2-^ produced and reduction of Fe^3+^ to Fe^2+^ by DT and thiourea, hence, some non-stoichiometric precipitates might be produced and resulted in some Fe–S type of magnetism impurities in S4 and S5. Meanwhile, the more through change of crystal growth habit in S5 might lead to more crystal defect, resulting in the strongest magnetism of S5.

### Luminescence mechanism of CuFe_2_S_3_ NCs

**Figure 8:**
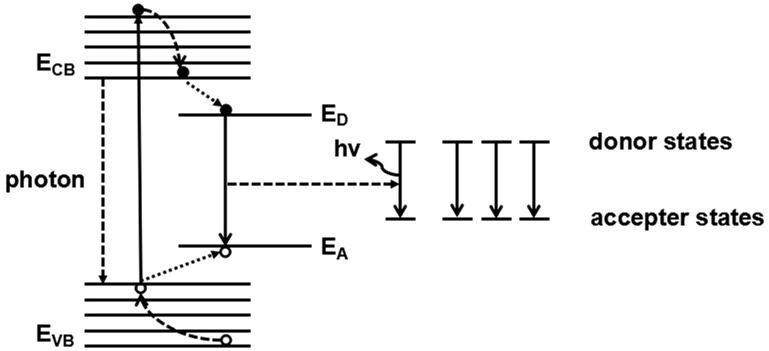
The luminescence mechanism of CuFe_2_S_3_ NCs

Similar with typical ternary sulfide such as CuInS_2_, the PL emission of CuFe_2_S_3_ NCs are also related to defect concentration. As schematically described in Fig. 8, in the ternary nano-crystalline band gap, there are many donor–acceptor states, usually from internal crystal defects [43–46]. Generally, the S vacancy and Cu interstitial ions act as the donors, while Cu vacancy and Fe interstitial ions act as the acceptors in the nanocrystals. When the Cu ions occupy the sites of Fe ions, or Fe ions occupy the positions of Cu ions, inverse defects are formed. Consequently, the excitons generated by the light absorption of CuFe_2_S_3_ NCs transform to these donor-acceptor-pair (DAP) defect states and recombine to give out emissions [47]. The enhanced luminescence from S1 to S5 should be related to the growing habit of the varied samples. Comparing with other samples, there is more obviously crystal deformation and defects in S5, due to the inhibitation of some crystal growth and atomic rearrangement, and this phenomenon results in a higher fluorescence emission.

These results reveal that by judiciously changing the composition of reactants and reaction conditions, uniform nano-sized cubic CuFe_2_S_3_ with near-infrared emission and magnetism could be obtained. The following cellular experiments proved that CuFe_2_S_3_ NCs have good biocompatibility and show great potential for cell labeling. The further application of this material to construct multifunctional system would be expected.

## Conclusions

In summary, a facile and mild one pot synthetic route was developed to synthesis CuFe_2_S_3_ NCs. Their phase composition and morphology, as well as magnetism and near-infrared fluorescence properties could be judiciously modulated by changing the precursors and surfactants. The mechanism related to these changes in physical and chemical properties were also discussed. The best magnetism and PL emission property are presented in cubic CuFe_2_S_3_ NCs with ‘nano-brick’ like morphology and well-ordered arrangement. Furthermore, nano-sized cubic CuFe_2_S_3_ was proved with good biocompatibility and near-infrared fluorescence properties, showing great potential for biological imaging. The synthesis of these nanoscale CuFe_2_S_3_ NCs with tunable composition, morphology and multiple properties would open a new avenue for directing the preparation of ternary chalcogenide nanocrystals with dual functions.
